# Phenotypic Plasticity of Leaf Shape along a Temperature Gradient in *Acer rubrum*

**DOI:** 10.1371/journal.pone.0007653

**Published:** 2009-10-29

**Authors:** Dana L. Royer, Laura A. Meyerson, Kevin M. Robertson, Jonathan M. Adams

**Affiliations:** 1 Department of Earth and Environmental Sciences, Wesleyan University, Middletown, Connecticut, United States of America; 2 Department of Natural Resource Sciences, University of Rhode Island, Kingston, Rhode Island, United States of America; 3 Tall Timbers Research Station, Tallahassee, Florida, United States of America; 4 Department of Biological Sciences, Rutgers University, Newark, New Jersey, United States of America; University of Sheffield, United Kingdom

## Abstract

Both phenotypic plasticity and genetic determination can be important for understanding how plants respond to environmental change. However, little is known about the plastic response of leaf teeth and leaf dissection to temperature. This gap is critical because these leaf traits are commonly used to reconstruct paleoclimate from fossils, and such studies tacitly assume that traits measured from fossils reflect the environment at the time of their deposition, even during periods of rapid climate change. We measured leaf size and shape in *Acer rubrum* derived from four seed sources with a broad temperature range and grown for two years in two gardens with contrasting climates (Rhode Island and Florida). Leaves in the Rhode Island garden have more teeth and are more highly dissected than leaves in Florida from the same seed source. Plasticity in these variables accounts for at least 6–19 % of the total variance, while genetic differences among ecotypes probably account for at most 69–87 %. This study highlights the role of phenotypic plasticity in leaf-climate relationships. We suggest that variables related to tooth count and leaf dissection in *A. rubrum* can respond quickly to climate change, which increases confidence in paleoclimate methods that use these variables.

## Introduction

Plant traits are determined by a combination of genetic heritage (genotype) and the capacity for responding to environmental change within a single genotype (phenotypic plasticity). Considerable attention has been given to unraveling the relative roles of plasticity and genotype in a wide array of leaf traits, in part to help understand how plants may respond to global climate change. For example, common garden experiments demonstrate that in most species both plastic and genetic factors are important for determining how photosynthetic efficiency, leaf area, and leaf mass per area respond to altitude [1]–[5]. Experimental treatments and other common garden experiments also typically find joint plastic-genetic control over how stomatal distributions, photosynthetic efficiency, leaf area, and/or leaf mass per area respond to irradiance [6], [7], temperature [8]–[10], water availability [9], and disturbance [11].

Leaf traits that can be measured in fossils are commonly used to reconstruct aspects of paleoclimate and paleoecology. For example, the site-mean leaf area among woody dicotyledonous taxa commonly scales with mean annual precipitation (MAP) [12], [13], and paleobotanists have applied this relationship for decades to reconstruct paleo-MAP [14]–[16]. The recognition that leaf area can exhibit a plastic response to environmental change is important for the paleobotanical community because it supports the required assumption that the measured trait values of a fossil reflect, without exception, the environment at the time of deposition [3], [17]. In other words, if the capacity for a trait to exhibit a plastic response were weak, trait values may instead reflect the environmental conditions of a variety of times prior to deposition; this time-lag effect may be particularly important during periods of rapid climate change [18], [19].

There are other leaf traits for reconstructing paleoenvironments for which the relative roles of plasticity and adaptation are unknown. Most notably, aspects of leaf teeth are commonly used as paleoenvironmental proxies. For example, the percentage of woody dicot species with leaf teeth at most sites worldwide inversely relates to mean annual temperature (MAT) [16], [20]–[25]. Recently, Huff et al. [26] and Royer et al. [27] found that site means of variables related to tooth count and tooth size also inversely relate to MAT. Additionally, Royer et al. [27] observed that the site mean of tooth area/leaf perimeter inversely correlates with leaf mass per area, thus opening the possibility for reconstructing leaf mass per area from fossils. The preponderance of teeth in colder climates is probably an adaptation to boost whole-plant carbon assimilation early in the growing season via the delivery of nutrients from enhanced sap flow [28] (see also refs. [29], [30]) and/or to prevent freeze-thaw embolisms via guttation [31]. Despite these advances towards clarifying the functional basis of leaf teeth, as well as their underlying genetic control [32]–[36], it is not clear how important phenotypic plasticity is for explaining many of the leaf-environment relationships observed by Huff et al. [26] and Royer et al. [27].

Royer et al. [27] also found that leaf dissection (e.g., perimeter: area ratio) inversely scales with MAT. A traditional explanation for this linkage, but in the opposite direction from the observations of Royer et al. [27], is that highly dissected leaves more efficiently shed heat [37]. Alternatively, highly dissected leaves (and including highly toothed leaves) can have high rates of transpiration [38], [39], which may be functionally linked to cold temperatures in the manner described in the previous paragraph. Some studies have investigated the phenotypic plasticity of leaf dissection. Gurevitch [40] discerned both a genetic and plastic component in the responses of leaf dissection to altitude in the herb *Achillea millefolium*. Similarly, phenotypic plasticity can partly explain the positive relationships that link nutrient availability to lobing in the tree *Crataegus monogyna* [41] and to leaf dissection in the aquatic herb *Sagittaria latifolia* [42]. Sack et al. [7] also reported a plastic component in the responses of leaf dissection to irradiance in three of six tree species; in contrast, Semchenko and Zobel [43] observed no plastic component in the responses of leaf lobation to irradiance in the herb *Serratula tinctoria*. Importantly, none of the above studies directly addressed temperature, a critical shortcoming for the paleobotanical community because aspects of leaf dissection are useful, along with other size and shape variables, for reconstructing climate [27].

As a first step towards investigating temperature in this context, Royer et al. [44] measured traits related to tooth count, tooth size, and leaf dissection in mature, native stands of two species with broad MAT ranges (*Acer rubrum* and *Quercus kelloggii*). Few correlations with MAT were found within *Q. kelloggii*, but within *A. rubrum* there were strong correlations that mirrored the across-species site-level patterns of Royer et al. [27]: plants growing in colder climates tend to have leaves that are more highly dissected and contain more teeth. Royer et al. [44] thus demonstrated that the leaf-climate relationships observed across species are also present within some (but not all) species. While the more limited genetic variability within species (compared to across species) suggests a role for plasticity in explaining these leaf-climate relationships, Royer et al. [44] could not exclude the possible influence of genetic differences across climatically-zoned ecotypes. Here, in an attempt to quantify directly how plasticity affects the responses of leaf teeth and leaf dissection to MAT, we measured the leaf shape of *A. rubrum* saplings that came from four climatically-distinct seed sources (MAT = 8.9−20.0°C) and were grown in two common gardens with contrasting MAT (9.8 and 20.0°C). Our goal was not to erect transfer functions for reconstructing climate from single species, but to use *A. rubrum* as a model species for exploring the relative influences of phenotypic plasticity and genotype on leaf shape variables that are pertinent for paleobotanists.

## Materials and Methods

### Seed sources and common gardens

**Figure 1:**
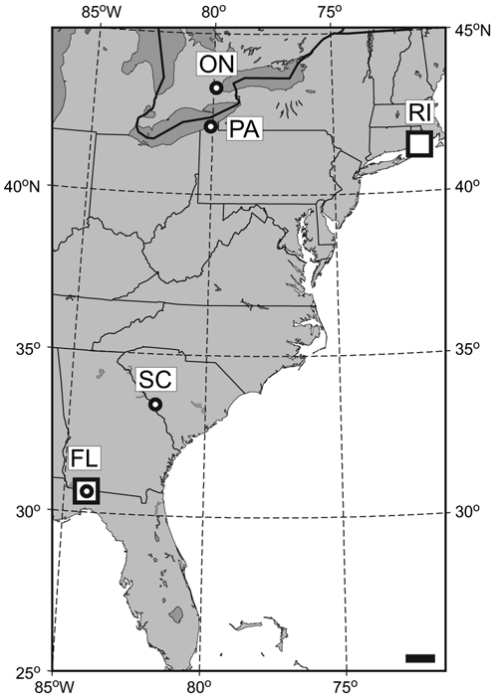
Map of seed collection sites (circles) and common gardens (large squares). ON = Burlington, Ontario; PA = Erie, Pennsylvania; RI = University of Rhode Island; SC = Savannah River, South Carolina; FL = Tall Timbers Research Station, Florida. Maps generated using GMT software (http://www.aquarius.ifm-geomar.de; Lambert azimuthal equal-area projection). Bar = 100 km.

Seed collection sites for *Acer rubrum* L. span an 11.1°C temperature gradient across the eastern U.S. and Canada (Fig. 1, Table 1). *A. rubrum* L. var. *rubrum* is present at the Ontario and Pennsylvania sites, while *A. rubrum* L. var. *trilobum* Torr. & A. Gray ex K. Koch is present at the two southern sites. These varieties are distinguished largely by morphology: *A. rubrum* var. *rubrum* produces more highly dissected leaves with more prominent teeth [45], [46]. Seeds were gathered from natural forests, rather than in plantations or yards where they may have a non-local origin. At each seed collection site, >10 seeds were collected from underneath each of >20 trees dispersed over 4 to 8 ha. Seeds were vernalized, planted in pots containing standard planting medium, and germinated in greenhouses near each garden in Spring 2006 prior to being transplanted to the gardens in July 2006.

**Table 1:** Summary of seed collection sites used in study.

Site name	Location	Elevation (m)	MAT (°C)	MAP (mm)	*n* (RI)	*n* (FL)
Burlington, Ontario	43.32°N, 79.88°W	150	8.9	879	21	3
Erie, Pennsylvania	42.12°N, 80.08°W	200	10.0	1086	33	5
Savannah River, South Carolina	33.45°N, 81.85°W	120	17.3	1132	38	13
Tall Timbers Research Station, Florida	30.65°N, 84.18°W	30	20.0	1606	29	15

Note.—MAT = mean annual temperature; MAP = mean annual precipitation; *n* (RI) = number of plants measured in Rhode Island garden; *n* (FL) = number of plants measured in Florida garden; climate data from stations with long-term records (>25 years; U.S. stations: U.S. National Climatic Data Center, http://www.ncdc.noaa.gov/oa/ncdc.html; Canadian station: Canadian Climate Data and Information Archive, http://www.climate.weatheroffice.ec.gc.ca/).

The two common gardens are in Rhode Island and Florida (Fig. 1). Both gardens are arranged in a grid pattern, with a 2.5×2.5 m spacing between plants; this spacing minimizes competition for above- and belowground resources, and facilitates site management in terms of mowing and weeding. Both gardens are on nearly level ground and are protected by deer fencing. *A. rubrum* shares space in the gardens with five other species not considered in the present study (*Liriodendron tulipifera*, *Quercus alba*, *Q. montana*, *Q. phellos*, *Q. rubra*). Plant assignments within the common gardens were random to control for microsite differences.

The Rhode Island garden occupies 1.6 ha of the Greene H. Gardner Crop Research Farm on the campus of the University of Rhode Island (41.40°N, 71.48°W, elevation = 22 m; MAT = 9.8°C; MAP = 1316 mm). Soils are Bridgehampton silt loam (Typic Dystrudept), a well-drained to moderately well-drained soil on glacial outwash plains and terraces. The garden is bordered on all sides by agricultural fields. Creeping red fescue (*Festuca rubra*) was planted as a ground cover to suppress weed growth. Irrigation was provided by an automated drip-line system with 0.6 m spacing.

The Florida garden is at the Tall Timbers Research Station near Tallahassee (30.65°N, 84.18°W; elevation = 30 m; MAT = 20.0°C; MAP = 1606 mm). The 2.5 ha garden is located in the middle of a 5 ha field that had been maintained with annual harrowing for purposes of wildlife management. The soil is somewhat poorly drained Albany loamy sand (Grossarenic Paleudult). Plants were watered regularly using a pumper truck, but in general they experienced more drought than plants in the Rhode Island garden, despite growing in a more poorly-drained soil. This drought factor partly explains the higher mortality of *A. rubrum* in Florida (74 vs. 29 % in Florida and Rhode Island gardens, respectively), but it probably does not affect our interpretations because water availability does not significantly impact variables related to tooth count, tooth size, and leaf dissection in *A. rubrum* [44].

### Leaf sampling and image analysis

Fully-expanded leaves were sampled at both gardens in October 2008. Plants thus experienced over two years of growth in their respective gardens. Across both gardens, 157 plants were sampled; for each seed source, 21–38 plants were sampled in the Rhode Island garden and, due to higher mortality, 3–15 in the Florida garden (Table 1). Because of the small stature (<2 m) and wide spacing (2.5 m) of the plants, all leaves are sun morphotypes.

**Figure 2:**
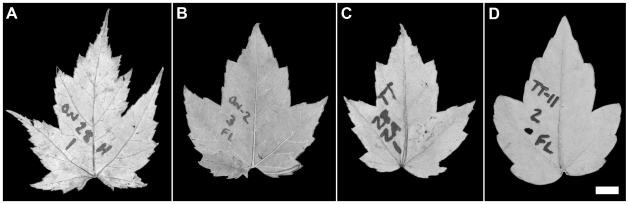
Representative leaves of *Acer rubrum* used in study. Leaf derived from Ontario seed stock grown in (A) Rhode Island and (B) Florida; leaf derived from Florida seed stock grown in (C) Rhode Island and (D) Florida. For all leaves, petioles have been removed and minor damage along the margin has been corrected with white lines. Bar (for all leaves) = 1 cm.

Leaves were dried, pressed, and photographed against a black velvet background (3264×2448 pixel resolution). Two leaves per plant were usually analyzed, although up to five leaves were used when there were <10 plants for a seed source × garden combination. Leaves were prepared for image analysis in Photoshop 10.0 (Adobe Systems, San Jose, CA, USA) following the protocols of Huff et al. [26] and Royer et al. [27]. In short, petioles are removed and any minor defects along the leaf margin are repaired using the line and eraser tools (e.g., Fig. 2A). After duplicating the prepared leaf, its teeth are detached; typically, this is done with a straight line between the bounding sinuses of each tooth (see ref. [27] for exceptions). When completed, there are two versions of the leaf: the complete leaf (with detached petiole) and the leaf with its teeth detached. Next, leaf size and shape variables that are mechanistically linked to MAT [28], [31] (see Introduction) are measured or calculated using Image-J (http://rsb.info.nih.gov/ij/). These variables can be grouped into three categories: leaf dissection (shape factor [4π × leaf area/perimeter^2^], compactness [perimeter^2^/area], perimeter ratio [perimeter/internal perimeter, where internal perimeter is the perimeter after the detachment of teeth]), tooth count (number of teeth, number of teeth/leaf perimeter, number of teeth/leaf area), and tooth size (tooth area, average area of a single tooth, tooth area/leaf perimeter, tooth area/leaf area). These are the same variables used by Huff et al. [26] and Royer et al. [27], [44]. For reference, highly dissected leaves have a low shape factor and a high compactness and perimeter ratio. All measured leaf variables are provided in Table S1 and leaf images are available by request from D.L.R.

### Statistics

Two-way analysis of variance (ANOVA) was used to test the influence of growth site and seed source on each leaf size and shape variable. Number of plants (*n* = 157) was the unit of replication for these tests. All size and shape variables are homoscedastic (Levene's Test of Equality of Error Variances; *P*>0.05) and normally distributed (one-sample Kolmogorov-Smirnov Test; *P*>0.05) after log-transforming five of the variables (compactness, number of teeth, number of teeth/leaf area, tooth area, and average area of a single tooth).

Separately, the partial *r^2^*-statistic from a multiple linear regression model was used to calculate the proportion of leaf size and shape variation within *A. rubrum* that can be explained by growth site and by seed source. For these models, the MATs of the growth site and seed source were the two independent variables and a leaf size or shape variable was the dependent variable. Growth site × seed source combinations was the unit of replication (*n* = 8). Multiple linear regression was used because correlations between MAT and leaf size and shape tend to be linear [27], [44] (see also Results).

## Results

**Figure 3:**
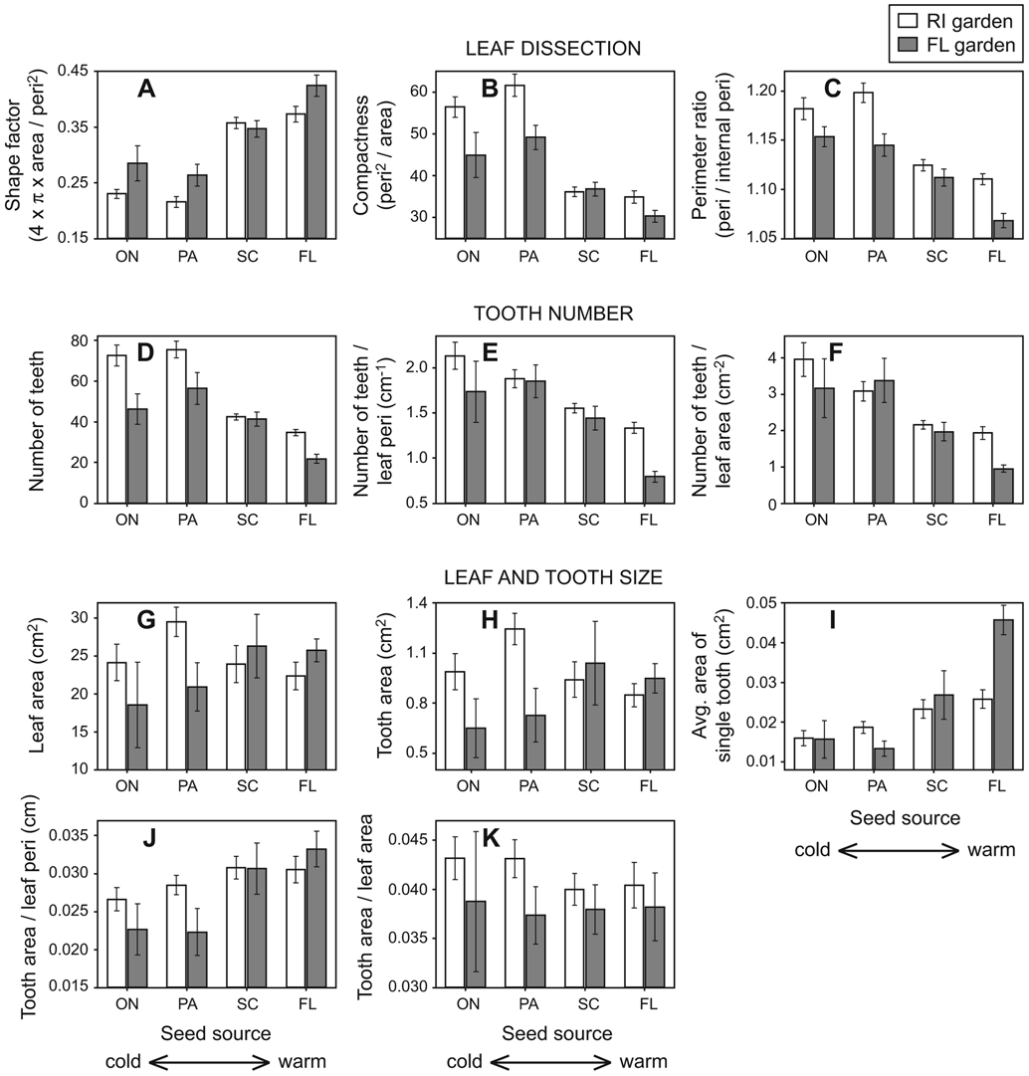
Leaf size and shape of *Acer rubrum*. Plants come from four different seed stocks and were grown in two gardens with contrasting climates. Variables are clustered into three groups: (A–C) leaf dissection; (D–F) tooth number, and (G–K) leaf and tooth size. ON, Ontario; PA, Pennsylvania; SC, South Carolina; FL, Florida; RI, Rhode Island; peri, perimeter. See Table 1, Figs. 1, and Materials and Methods for details about seed collection sites and gardens. Differences between gardens reflect phenotypic plasticity, while differences among seed stock probably mostly reflect genetic differences among ecotypes. Standard errors are plotted.

There are many strong differences in leaf shape between the two gardens and among the four seed sources. In general, plants growing in the colder Rhode Island garden and seeds native to colder climates produce leaves that have more teeth (Figs. 2, 3D–F) and are more highly dissected (Figs. 2, 3A–C; low shape factor, high compactness and perimeter ratio). When growth site and seed source are scrutinized independently from one another using two-way analysis of variance, the influence of both factors on tooth count and leaf dissection remain statistically significant (Table 2). Importantly, there are no statistically-significant (α = 0.05) interactions between growth site and seed source except for average area of a single tooth (*P* = 0.006; Table 2).

**Table 2:** Summary of statistics used to test the influence of growth site (phenotypic plasticity) and seed source (ecotypic variation) on leaf size and shape in *Acer rubrum*.

	Growth site		Seed source		Growth × seed	
Variable	*F_[1, 149]_*	*P*	*F_[3, 149]_*	*P*	*F_[3, 149]_*	*P*
Leaf dissection						
Shape factor	6.7	**0.011**	34.3	**<0.001**	2.0	0.11
Compactness	8.5	**0.004**	33.9	**<0.001**	2.0	0.12
Perimeter ratio	13.7	**<0.001**	21.4	**<0.001**	1.3	0.27
Tooth number						
Number of teeth	21.4	**<0.001**	40.8	**<0.001**	2.5	0.06
Number of teeth / leaf perimeter (cm^−1^)	6.8	**0.01**	18.1	**<0.001**	2.0	0.11
Number of teeth / leaf area (cm^−2^)	4.5	**0.04**	21.8	**<0.001**	1.3	0.28
Leaf and tooth size						
Leaf area (cm^2^)	0.61	0.44	0.32	0.81	1.3	0.26
Tooth area (cm^2^)	2.8	0.10	0.23	0.88	2.3	0.08
Average area of a single tooth (cm^2^)	0.94	0.33	15.1	**<0.001**	4.4	**0.006**
Tooth area / leaf perimeter (cm)	0.91	0.34	3.5	**0.02**	1.2	0.32
Tooth area / leaf area	2.1	0.15	0.12	0.95	0.15	0.93

Note.—Variables are grouped by relatedness to leaf dissection, tooth number, or leaf and tooth size. Two-way analysis of variance (ANOVA) used for all tests; ‘growth site’ and ‘seed source’ test for no differences in a leaf variable between the two sites of growth and among plants with different seed sources, respectively; ‘growth × seed’ tests for no differences in a leaf variable due to the interaction between growth site and seed source; outcomes indicating significant differences (*P*<0.05) are in bold.

Leaf size can affect the number of teeth, although in our data set leaf area does not vary significantly between growth sites or among seed sources (Fig. 3G; Table 2). Nonetheless, after taking leaf size into account, the influence of seed source and growth site on tooth count remains statistically significant (‘number of teeth/leaf peri’ and ‘number of teeth/leaf area’; Figs. 3E–F; Table 2). Of the total measured variance in the statistically-significant leaf-shape variables in Table 2, growth site alone explains 6–19 % of the variance while seed source explains 65–87% (Table 3).

**Table 3:** Proportion of leaf size and shape variation within *Acer rubrum* that can be explained by growth site (phenotypic plasticity) and seed source (ecotypic variation).

Variable	Growth site (*r^2^*)	Seed source (*r^2^*)
Leaf dissection		
Shape factor	**0.06**	**0.87**
Compactness	**0.11**	**0.77**
Perimeter ratio	**0.19**	**0.75**
Tooth number		
Number of teeth	**0.19**	**0.69**
Number of teeth/leaf perimeter (cm^−1^)	**0.12**	**0.75**
Number of teeth/leaf area (cm^−2^)	**0.05**	**0.87**
Leaf and tooth size		
Leaf area (cm^2^)	0.11	0.05
Tooth area (cm^2^)	0.22	0.01
Average area of a single tooth (cm^2^)	0.05	**0.65**
Tooth area/leaf perimeter (cm)	0.06	0.72
Tooth area/leaf area	0.73	0.11

Note.— Variables are grouped by relatedness to leaf dissection, tooth number, or leaf and tooth size. Results for each leaf size and shape variable are based on a multiple linear regression model (see Materials and Methods for details); *r^2^*-statistic reflects the individual (partial) contribution of growth site or seed source; *r^2^* of the full model is the sum of the two partials. For convenience, significant effects from Table 2 (*P*<0.05) are in bold.

In contrast to tooth count and leaf dissection, growth site does not significantly influence any variables related to tooth size (Fig. 3H–K; Table 2). Similarly, seed source does not significantly influence tooth area or tooth area/leaf area, but the average area of a single tooth and tooth area/leaf perimeter are more likely to be larger in plants with a warmer seed source (Fig. 3H–K; Table 2).

## Discussion

After two years of growth, site of growth has an impact on the leaf shape of *A. rubrum* that is independent of genetic stock. In short, plants growing in the colder Rhode Island garden produce leaves that have more teeth and are more highly dissected than genetically-similar plants grown in Florida (Figs. 2, 3A–F; Table 2); moreover, these patterns mostly hold regardless of seed source (Table 2). These results reveal an important source of phenotypic plasticity within *A. rubrum* that covaries with MAT in a predictable manner. The patterns are also consistent with studies that find a functional link between leaf teeth and cold climates [28], [31] (see Introduction). Regression analyses indicate that phenotypic plasticity, as represented by growth site, can explain 5–19% of the variance observed for variables related to tooth count and leaf dissection (Table 3). Given that two years of growth may be too short to generate a full response of phenotypic plasticity to a one-step change in environment, these values in our opinion represent minima.

**Figure 4:**
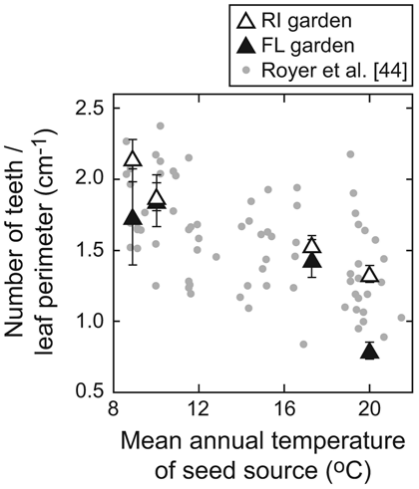
Sensitivity of number of teeth/leaf perimeter to mean annual temperature in *Acer rubrum*. RI = Rhode Island; FL = Florida. See Fig. 1 and Materials and Methods for details about gardens. Differences within a single seed source reflects plasticity within a genotype; differences within a single garden, and within the Royer et al. [44] data set, probably mostly reflect genetic differences among ecotypes. Garden data are identical to Fig. 3E.

An even larger share of the variance in tooth count and leaf dissection variables can be explained by seed source (69–87%; Table 3), with plants from colder seed sources producing more highly dissected leaves with more, but smaller teeth (Table 2). This variance likely reflects differences among genetically-distinct *A. rubrum* populations. Thus, we detect both plastic and genetic responses to climate for leaf traits related to tooth count and degree of dissection. In combination with Royer et al. (2008), who observed continuous gradients in leaf shape across MAT and different ecotypes (e.g., Fig. 4), our results suggest that the shape differences used to discriminate between *A. rubrum* var. *rubrum* (Pennsylvania and Ontario seed stock) and *A. rubrum* var. *trilobum* (Florida and South Carolina seed stock) can be partly explained by phenotypic plasticity. The magnitudes of the plastic responses across different seed sources are broadly similar, although they tend to be weakest in plants from the South Carolina seed stock (Fig. 3), signaling a possible genetic gradient in plasticity. However, across our whole sample, there are no statistically significant interactions between growth site and seed source for our size and shape variables with the exception of average area of a single tooth.

The leaf-climate relationships observed here largely match that of Royer et al. [44] (e.g., Fig. 4), who measured the responses of leaf size and shape to MAT in mature *A. rubrum* populations along the U.S. east coast (from northern Vermont to southern Florida). Royer et al. [44] found that plants native to colder climates have more dissected leaves with more, but smaller teeth; moreover, they found no significant relationship between MAT and tooth area/leaf area. The congruence in patterns between these two studies suggests that potential confounding factors in the current study (e.g., differences in water availability between gardens, using saplings instead of mature plants) were probably not significant.

The biggest contrast between the studies is leaf area: we find no correlation between leaf area and MAT, while Royer et al. [44] observed a significant negative relationship (*r^2^* = 0.38; *P*<0.001; *n* = 77; least-squares linear regression). It is not clear what underlies this difference: if inadequate access to water were important at the Florida site, one would expect smaller leaves [12], [13], [15], [16]. However, in both studies, tooth area strongly tracks leaf area (e.g., Fig. 3G–H); after taking into account leaf size, the impact of MAT on tooth size is minimal in both studies (‘tooth area/leaf peri’ and ‘tooth area/leaf area’; Figs. 3J–K; Table 2) [44]. The similarity across studies in these latter responses also speaks to a general and consistent relationship between leaf teeth and climate in *A. rubrum*. The lack of a strong plastic component in the tooth area responses suggests that tooth size variables may respond more slowly to periods of rapid temperature change; this caveat should be considered when interpreting paleotemperature estimates based on such variables.

### Concluding remarks

Our study demonstrates that variables related to leaf dissection and tooth number in *A. rubrum* respond plastically to their environment. Our results are consistent with other studies that find both plastic and genetic responses to environmental change in other leaf traits such as photosynthetic performance and leaf mass per area (see Introduction). Together, these studies indicate that leaf traits in many species can respond quickly to environmental change.

This is welcome news for paleobotanists who measure leaf traits in fossils as proxies for paleoclimate and paleoecology; in particular, leaf teeth are central to several paleoenvironment proxies [16], [24], [27]. Our results thus support the tacit assumption that fossil leaf traits faithfully reflect the environmental conditions at the time of deposition, and increase the confidence in paleoclimate reconstructions based on leaf size and shape. However, it is important to acknowledge that plasticity is but one process that determines the distribution of leaf traits at a fossil locality; other processes, which all operate on slower timescales, may include evolutionary changes within populations and the dispersal of ecotypes and species. Also, single localities in different depositional settings may contain fossils deposited over varying intervals of time, ranging from instantaneous to 10^2^–10^3^ years [47]. Thus, the importance of plasticity at a given fossil locality may be subordinate to these other factors; indeed, in our more limited extant study, plasticity accounts for 5–19 % of trait variation, although this is probably an underestimate (see earlier discussion). In addition, it is not known how other environmental factors that may have varied greatly over geological timescales such as atmospheric CO_2_ [48], [49] and UV-intensity impact our measured traits. Nevertheless, the recognition that leaf teeth respond plastically to MAT in a predictable fashion increases confidence that individual fossil localities render accurate, temporally-resolved snapshots of paleoenvironments.

## Supporting Information

Table S1 Size and shape data for all leaves used in study
(0.02 MB TXT)
